# Overall survival and second primary malignancies in men with metastatic prostate cancer

**DOI:** 10.1371/journal.pone.0227552

**Published:** 2020-02-21

**Authors:** Juha Mehtälä, Jihong Zong, Zdravko Vassilev, Gunnar Brobert, Montse Soriano Gabarró, Pär Stattin, Houssem Khanfir

**Affiliations:** 1 EPID Research, Espoo, Finland; 2 Bayer LLC, Whippany, New Jersey, United States of America; 3 Bayer AB, Stockholm, Sweden; 4 Bayer AG, Berlin, Germany; 5 Department of Surgical Sciences, Uppsala University, Uppsala, Sweden; Istituto Scientifico Romagnolo per lo Studio e la Cura dei Tumori (IRST) - IRCCS, ITALY

## Abstract

**Background:**

Among prostate cancer (PC) patients, over 90% of distant metastases occur in the bone. PC treatments may be associated with side effects, including second primary malignancies (SPM). There is limited information on the incidence of SPM among men with bone metastatic PC (mPC) and among men with bone metastatic castration-resistant PC (mCRPC). We estimated overall survival and the incidence of SPM in men with mPC and mCRPC.

**Methods:**

In the Prostate Cancer data Base Sweden, the National Prostate Cancer Register was linked to other national health care registers, 15,953 men with mPC in 1999–2011 were identified. Further, 693 men with mCRPC were identified. Outcomes were evaluated using stratified incidence rates, Kaplan-Meier estimators and Cox models.

**Results:**

The mean age among men with mPC was 73.9 years and in men with mCRPC 70.0 years. The median respective survivals were 1.5 (13,965 deaths) and 1.14 years (599 deaths), and average times since PC diagnosis 1.8 and 4.7 years. We observed 2,669 SPMs in men with mPC and 100 SPMs in men with mCRPC. The incidence rate of SPM per 1,000 person-years was 81.8 (78.8–85.0) for mPC and 115.6 (95.1–140.7) for mCRPC. High age, prior neoplasms, urinary tract infection, congestive heart failure, diabetes and renal disease were most strongly associated with increased mortality risk. Prior neoplasms and prior use of antineoplastic agents were most strongly associated with increased SPM risk. Several factors associated with increased mortality and SPM risks were more prevalent in the mCRPC cohort.

**Conclusions:**

Our results on mortality for men with mPC and mCRPC are in line with previous studies from the same time period. Investigation of factors associated with mortality and SPM in men with mPC and mCRPC can help to further understand these outcomes in the era prior to several new treatments have come available.

## Introduction

A great threat to survival and quality of life for men with prostate cancer (PC) is posed by development of bone metastases[1]. More than 90% of distant metastases occur in the bone[2,3]. Therefore, nearly all treatments for metastatic PC are directed towards eradicating or limiting bone metastases or palliating their side effects[4]. Once PC becomes metastatic, the survival of the patient depends on the extent of the metastatic burden, response to therapy and the site of metastases[5–9].

PC cells are stimulated by androgens[10] and consequently androgen deprivation therapy (ADT) is the treatment of choice for men with PC. ADT decreases androgen levels and thereby slows down progression of the disease[11,12]. The effectiveness of ADT wanes over time and disease progression continues despite low testosterone levels[13]. At this point the disease is referred to as castration-resistant prostate cancer (CRPC). Development of CRPC to bone metastatic CRPC (mCRPC) can be determined by bone scan and is associated with pain and risk of pathological fractures notably in the spine, pelvis and hip[14].

CRPC is a heterogeneous disease and its exact definition is not long-established. A proposal to standardize the CRPC definition has been based on combination of serum castration levels of testosterone, rises in prostate-specific antigen (PSA) levels, ADT withdrawal and progression of osseous or soft tissue lesions[15,16]. In practice, however, epidemiological studies have used various methods to identify patients with CRPC[14].

Among other factors, response to therapy, serum PSA levels, time to progression, and development of metastases affect prognosis among patients with CRPC[5–9,17,18]. In epidemiological studies, data on clinical parameters affecting prognosis and (m)CRPC definition is varying. This in part, may have affected the variation in reported median survival across different studies, ranging from 9 to 30 months in CRPC and mCRPC patients [14,19–23]. Additionally, overall survival has been increasing with the introduction of newer hormonal agents in recent years (after 2011) such as abiraterone[24] and enzalutamide[7].

PC treatments may have side effects, including second primary malignancies (SPM) after radiation therapy[25,26]. To date, an increased risk of SPM following radiation therapy for PC has been reported[27,28], but there is no information on the incidence of SPM among men with CRPC or mCRPC. As physicians need to evaluate the risk-benefit ratio in treatment selection, there is a need to provide more information on SPM among these patients.

The primary aim of this study was to estimate overall survival and the overall incidence rate of SPM in patients with bone metastatic PC (mPC) and mCRPC. The secondary aim was to assess site-specific SPM incidence rates among these patients. Both aims were descriptive, and the intention was not to perform a direct comparison between the two cohorts. The results of this study may allow for indirect comparison to SPM rates identified in mPC and mCRPC populations across other studies. Nordic nationwide population-based registers containing treatment information, hospital diagnoses, times and causes of death among other relevant information, collected comprehensively for all patients as part of the daily administrative routines, provide a good data source for providing such information.

## Materials and methods

In this register-based cohort study, data were extracted from the Prostate Cancer data Base Sweden (PCBaSe) which links the National Prostate Cancer Register (NPCR) of Sweden to other national health care registers and demographic databases.

NPCR contains comprehensive data including cancer characteristics at the time of PC diagnosis. In addition, PCBaSe includes information on diagnoses of disease outcomes and comorbidities by use of data from the National Patient Register (NPR), Swedish Cancer Registry (SCR), filled prescriptions on drugs by use of data in The Prescribed Drug Register (PDR), and cause of death from The Cause of Death Register (CDR) and immigration, emigration information from the Population Register (PR)[29].

### Study populations and specific data sources

The primary aim of this study was to estimate overall survival and the incidence of SPM in two study populations. First, men diagnosed with bone metastatic PC (mPC cohort), and second, a subset of men with mPC fulfilling conditions for castration resistant prostate cancer (mCRPC cohort). Inclusion criteria for the mPC cohort were PC diagnosis in 1 January 1998–31 December 2011, and first bone metastasis diagnosis in 1 January 1999–31 December 2011, identified by the use of data in the NPR, NPCR, or SCR (detailed diagnosis codes are presented in Table A in S1 File). The mCRPC cohort was identified within the group of mPC patients by their use of drugs to treat CRPC (mitoxantrone, estramustine, ketoconazole, docetaxel, cabazitaxel, abiraterone, enzalutamide; Table A in S1 File) in 1 January 2006–31 December 2013, identified from the NPR or the PDR. Data on drugs in PDR are available from July 2005, and in the NPR since 1987, however recording of inpatient drugs is not mandatory and capture rate is low.

Pre-specified exclusion criteria were PC diagnosis later than 2 months after the diagnosis of first bone metastases, permanent residence not being in Sweden for at least a year before cohort entry (based on data on immigration / emigration in the PR), or if patient had used any radiopharmaceuticals for bone metastases at any time.

### Cohort entry date and follow-up

Cohort entry for mPC was defined as the date of the first bone metastasis diagnosis. For the mCRPC cohort, cohort entry was defined as the date of first CRPC-specific therapy. Follow-up started at cohort entry and ended at death, at the end of the study period (31 December 2013), or at emigration from Sweden, whichever occurred first. In the analyses of SPM, follow-up was discontinued also after the first outcome event. This set-up allowed a minimum of 2 year of potential follow-up for each patient.

### Outcomes

The primary outcomes in this study were SPM and death. Secondary objective was to identify site-specific SPMs (all solid tumors combined, bladder cancer, rectum cancer, colon cancer, lung cancer, myelodysplastic syndrome, and leukemia). Detailed definitions of all outcomes are presented in Table A in S1 File.

Primary cancer of bone and cartilage was omitted from the outcomes due to very high number of observed cases (8%; 169 of 2049 solid tumor cases). The most likely reason for these numbers was that pre-existing bone metastases from PC were misclassified as a primary cancer.

### Other variables

Several comorbidities including history of other cancers, urinary tract infection, hypertension, congestive heart failure, hyperlipidemia, diabetes mellitus, renal disease and liver disease were summarized at baseline and used in stratified and multivariate analyses. In addition, received therapies were investigated. Detailed definitions of these variables are presented in Table A in S1 File.

### Main statistical methods

We calculated incidence rates of SPMs and mortality with total follow-up time (person-years), number of events and the 95% confidence interval (CI). For overall survival, we also calculated the Kaplan-Meier estimates. Age was the main stratifying variable when calculating incidence rates. Additional stratifying variables were given therapies and comorbid conditions. Hazard ratios (HR) were estimated using multivariate Cox regression and used to describe adjusted effects of risk factors on the overall survival and on the incidence of SPMs.

### Study conduct

The study was conducted according to an observational study protocol developed following the guidance and methodological standards of the European Network of Centres for Pharmacoepidemiology and Pharmacovigilance (ENCePP). The study protocol is registered and available at the ENCePP E-Register of Studies no EUPAS21285. This study was approved by the regional ethics committee in Stockholm (Regionala etikprövningsnämnden i Stockholm—diary number: 2016/443-31/2).

## Results

From 56,515 patients diagnosed with PC in 1998–2011, we identified 15,995 who had their first bone metastasis diagnosis in 1999–2011. After applying the exclusion criteria, 15,953 patients were included in the mPC cohort. For the mCRPC cohort, 693 patients were included, this being 7% of all potential mPC patients with cohort entry in 2006 or later. Ten patients whose follow-up ended at the same day as their follow-up started were excluded from the outcome analyses in the mPC cohort, leaving 15,943 patients. In the mCRPC cohort, all 693 patients had at least one day of follow-up, and were all included in the outcome analysis.

Baseline characteristics of patients in each of the two cohorts are presented in Table 1. The mean (median, range) ages in the mPC and mCRPC cohorts were 73.9 (75, 37–101), 70.0 (70, 42–91), respectively. The respective average times since PC diagnosis were 1.8 and 4.7 years. In the mPC cohort, 62% had bone metastases already at the initial PC diagnosis, which in the mCRPC cohort did not happen due to the cohort definition. Nearly all comorbidities were more prevalent in the mCRPC cohort, including prior malignant neoplasms (17% vs. 13%), prior secondary malignant neoplasms (15% vs. 5%), renal disease (17% vs. 13%). The mCRPC cohorts also had more frequent history of prior treatments, including radiation therapy (19% vs. 5%). In both cohorts, the most commonly used ADTs prior to cohort entry were bicalutamide, leuprorelin, and goserelin (Table 1). In the mCRPC cohort, the most common CRPC treatments used after bone metastases were estramustine (68%) and ketoconazole (47%). Given the study period, the use of the newer hormonal therapies was very limited (Table B in S1 File).

**Table 1:** Patient characteristics among prostate cancer patients with bone metastases (mPC), and among castration-resistant prostate cancer patients with bone metastases (mCRPC). All variables, excluding duration of follow-up, are presented at cohort entry.

Variable	mPC cohort	mCRPC cohort
Age (years)		
Mean (SD)	73.9 (8.9)	70.0 (8.2)
Median (Q1, Q3)	75 (68, 81)	70 (65, 76)
Min–Max	37–101	42–91
Cohort entry year		
≤ 2006^1^	7510 (47.08%)	127 (18.33%)
2007–2009	4713 (29.54%)	241 (34.78%)
≥ 2010^2^	3730 (23.38%)	325 (46.90%)
Time since first PC diagnosis (years)^3^		
Mean (SD)	1.81 (3.02)	4.73 (2.86)
Median (Q1, Q3)	0 (0, 2.97)	4.22 (2.40, 6.48)
Min–Max	-0.16–13.64	0.47–13.74
Duration of follow-up at the end of study (days)		
Mean (SD)	843 (891)	499 (439)
Median (Q1, Q3)	556 (210, 1141)	379 (187, 662)
Min–Max	0–5460	3–2821
Prior malignant neoplasm	2082 (13.05%)	116 (16.74%)
Prior secondary malignant neoplasm	833 (5.22%)	103 (14.86%)
Urinary tract infection	1616 (10.13%)	110 (15.87%)
Retention of urine	2673 (16.76%)	144 (20.78%)
Abnormal serum enzyme levels	2242 (14.05%)	94 (13.56%)
Unspecified haematuria	1627 (10.20%)	86 (12.41%)
Liver disease	107 (0.67%)	11 (1.59%)
Renal disease	2066 (12.95%)	119 (17.17%)
Hypertension	3835 (24.04%)	178 (25.69%)
Angina pectoris	1979 (12.41%)	73 (10.53%)
Congestive heart failure	1516 (9.50%)	32 (4.62%)
Diabetes mellitus	1553 (9.73%)	65 (9.38%)
Hyperlipidaemia	1025 (6.43%)	42 (6.06%)
Prior ADT use (any)	5186 (32.51%)	643 (92.78%)
Prior bicalutamide use	4191 (26.27%)	572 (82.54%)
Prior flutamide use	446 (2.80%)	79 (11.40%)
Prior nilutamide use	4 (0.03%)	1 (0.14%)
Prior leuprorelin use	3076 (19.28%)	359 (51.80%)
Prior goserelin use	1187 (7.44%)	206 (29.73%)
Prior antineoplastic or immunomodulating agent use	5262 (32.98%)	643 (92.78%)
Prior endocrine therapy	5180 (32.47%)	643 (92.78%)
Bilateral orchiectomy performed in history	851 (5.33%)	54 (7.79%)
Radical prostatectomy performed in history	472 (2.96%)	69 (9.96%)
Prior radiation therapy	736 (4.61%)	132 (19.05%)
Prior prednisolone use	1510 (9.47%)	400 (57.72%)
Prior opioid use	5238 (32.83%)	474 (68.40%)

^1^Minimum cohort entry year is 1999 in mPC cohort and 2006 in mCRPC cohort

^2^Maximum cohort entry year is 2011 in mPC cohort and 2013 in mCRPC cohort

^3^PC diagnosis must be prior to cohort entry or at most 2 months after allowing the minimum to be negative

**Fig 1:**
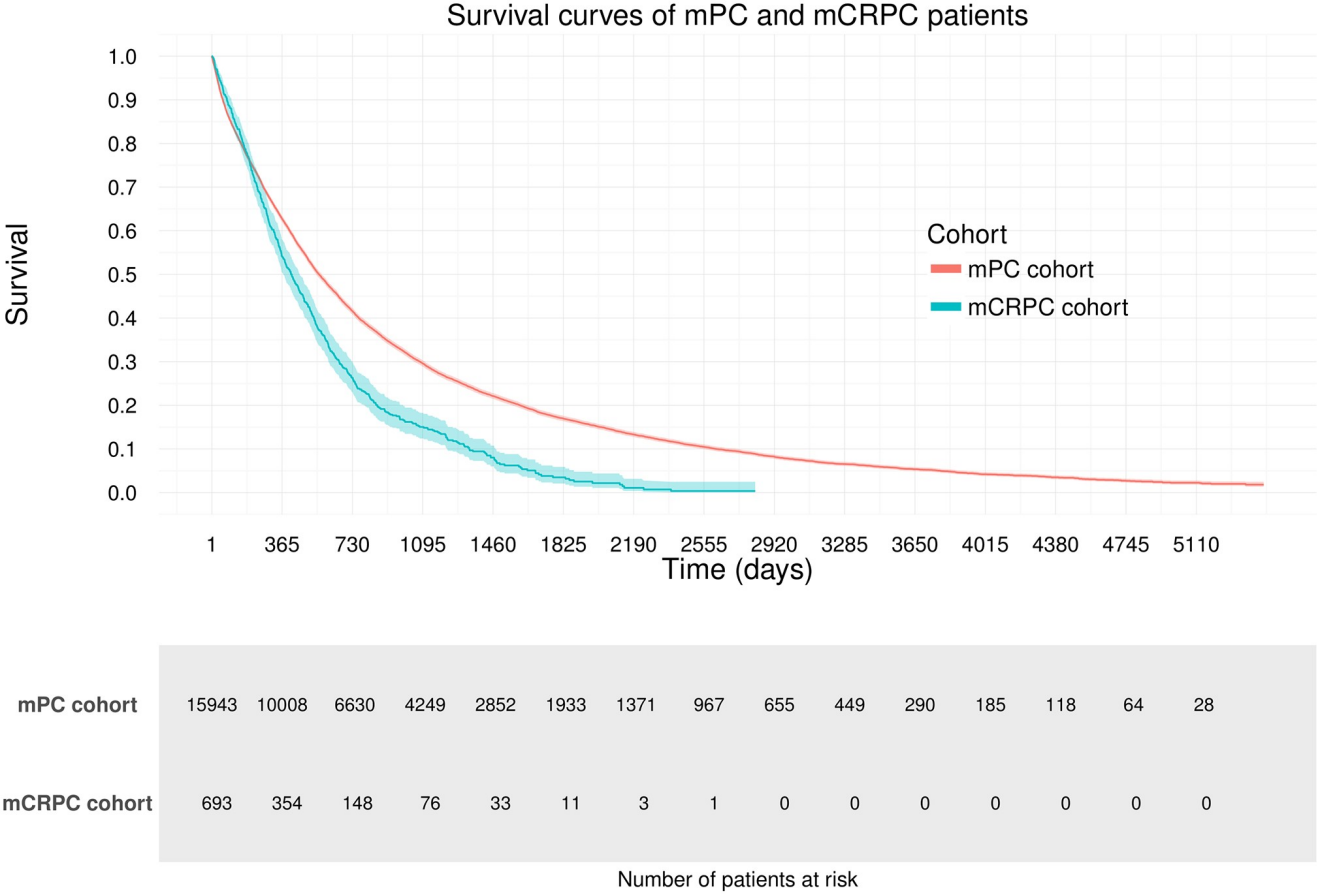
Kaplan-Meier survival curves among prostate cancer patients with bone metastases (mPC), and among castration-resistant prostate cancer patients with bone metastases (mCRPC).

The median survival in men with mPC was 1.5 years (13,965 deaths; 1^st^ quartile 0.6 years, 3^rd^ quartile 3.6 years, minimum 1 day, maximum >15 years). In the first mCRPC cohort identified using treatment specific to mCPRC, median survival was 1.1 years (599 deaths). The Kaplan-Meier survival plots for the two cohorts are presented in Fig 1. The respective mortality rates among mPC and mCRPC cohorts were 379.4 (95% CI: 373.2–385.8) and 632.8 (95% CI: 584.1–685.6) per 1,000 person-years (Tables 2 and 3).

**Table 2:** Total and stratified mortality and second primary malignancy (SPM) rates with adjusted hazard ratios (HR) among prostate cancer patients with bone metastases (mPC).

Mortality					SPM			
Variable	Events	PY	IR^1^ (95% CI)	HR^2^ (95% CI)	Events	PY	IR^1^ (95% CI)	HR^2^ (95% CI)
**mPC cohort (Total)**	13,965	36,806	379.43 (373.18, 385.77)	-	2,669	32,623	81.81 (78.77, 84.98)	-
**Age**								
<65	1,510	5,169	292.12 (277.75, 307.23)	reference	392	4,727	82.93 (75.11, 91.56)	reference
65–69	1,640	5,472	299.68 (285.52, 314.54)	1.00 (0.94, 1.06)	398	4,903	81.17 (73.58, 89.55)	0.97 (0.85,1.10)
70–74	2,243	6,915	324.36 (311.21, 338.07)	1.08* (1.02, 1,15)	504	6,166	81.74 (74.91, 89.20)	1.06 (0.94, 1.19)
75–79	2,974	7,926	375.20 (361.95, 388.93)	1.32† (1.25, 1.40)	605	6,977	86.71 (80.07, 93.91)	1.04 (0.92, 1.17)
80–84	3,055	6,762	451.79 (436.05, 468.10)	1.73† (1.63, 1.84)	480	5,916	81.13 (74.19, 88.72)	1.01 (0.88, 1.15)
>84	2,543	4,560	557.61 (536.36, 579.71)	2.23† (2.08, 2.40)	290	3,933	73.73 (65.71, 82.72)	0.97 (0.82, 1.16)
**Cohort entry year**								
1999–2002	3,511	11,840	286.15 (276.68, 295.95)	reference	706	10,470	67.43 (62.64, 72.60)	reference
2003–2006	3,997	11,380	321.52 (311.27, 332.11)	0.94* (0.89, 0.98)	789	9,892	79.76 (74.39, 85.53)	1.13* (1.01, 1.25)
2007–2009	4,707	8,350	494.11 (479.26, 509.42)	1.03 (0.98, 1.09)	701	7,454	94.04 (87.33, 101.27)	1.13* (1.00, 1.27)
2010–2011	3728	5,235	533.33 (513.91, 553.49)	0.94 (0.89, 1.00)	473	4,807	98.40 (89.92, 107.68)	1.06 (0.93, 1.21)
**Prior malignant neoplasm**								
Yes	3,238	5,170	626.27 (605.06, 648.21)	1.16† (1.09, 1.23)	373	2,767	134.80 (121.79, 149.20)	1.44† (1.27, 1.62)
No	10,727	31,635	339.08 (332.73, 345.56)	reference	2,296	29,856	76.90 (73.82, 80.11)	reference
**Prior secondary malignant neoplasm**								
Yes	2,236	1,416	1,579.22 (1,515.11, 1,646.06)	2.21† (1.97, 2.48)	239	1,748	136.76 (120.48, 155.25)	1.57† (1.27, 1.95)
No	11,729	35,390	331.42 (325.48, 337.48)	reference	2430	30,875	78.70 (75.64, 81.90)	reference
**Urinary tract infection**								
Yes	3,785	4,284	815.09 (788.50, 842.58)	1.29† (1.21, 1.38)	361	3,600	100.27 (90.45, 111.17)	1.09 (0.94, 1.27)
No	14,328	32,521	322.03 (315.92, 328.26)	reference	2,308	29,023	79.52 (76.35, 82.84)	reference
**Hypertension**								
Yes	4,816	9,604	501.46 (487.50, 515.82)	1.06* (1.01, 1.11)	764	8,157	93.66 (87.25, 100.54)	1.01 (0.91, 1.13)
No	9,149	27,202	336.34 (329.52, 343.30)	reference	1,905	24,466	77.86 (74.45, 81.44)	reference
**Congestive heart failure**								
Yes	2,661	3,516	756.73 (728.52, 786.04)	1.42† (1.33, 1.53)	286	2,988	95.72 (85.25, 107.48)	1.11 (0.95, 1.30)
No	11,304	33,289	339.57 (333.37, 345.89)	Reference	2,383	29,635	80.41 (77.25, 83.71)	reference
**Hyperlipidaemia**								
Yes	1,111	2,571	432.11 (407.43, 458.28)	0.96 (0.89, 1.04)	216	2,176	99.27 (86.88, 113.44)	1.16 (0.99, 1.37)
No	12,854	34,234	375.47 (369.03, 382.02)	reference	2,453	30,447	80.57 (77.44, 83.82)	reference
**Diabetes mellitus**								
Yes	2,052	3,881	528.74 (506.35, 552.11)	1.19† (1.11, 1.27)	315	3,288	95.80 (85.78, 106.98)	1.00 (0.87, 1.16)
No	11,913	32,925	361.83 (355.39, 368.38)	reference	2,354	29,335	80.25 (77.07, 83.55)	reference
**Renal disease**								
Yes	3,408	4,768	714.74 (691.14, 739.14)	1.26† (1.19, 1.34)	425	4,047	105.01 (95.48, 115.48)	0.98 (0.85, 1.12)
No	10,557	32,037	329.52 (323.29, 335.87)	reference	2,244	28,575	78.53 (75.35, 81.85)	reference
**Liver disease**								
Yes	177	272	650.72 (561.58, 754.01)	0.95 (0.73, 1.22)	32	229	139.89 (98.92, 197.81)	1.58* (1.03, 2.41)
No	13,788	36,534	377.41 (371.16, 383.76)	reference	2,637	32,394	81.40 (78.36, 84.57)	reference
**Radiation therapy**								
Yes	2,862	3,712	770.95 (743.22, 799.72)	1.02 (0.92, 1.12)	345	3,089	111.68 (100.49, 124.10)	1.15 (0.93, 1.41)
No	11,103	33,093	335.51 (329.32, 341.80)	reference	2,324	29,533	78.69 (75.56, 81.96)	reference
**Antineoplastic agents**								
Yes	1,024	1,309	782.03 (735.57, 831.43)	1.27* (1.08, 1.51)	147	1,014	145.00 (123.36, 170.44)	1.72† (1.32, 2.24)
No	12,941	35,496	364.57 (358.35, 370.91)	reference	2,522	31,609	79.79 (76.73, 82.96)	reference

^1^ Incidence rate (IR) per 1,000 patient years (PY)

^2^ Cox’s proportional hazard model included the following time-independent variables (status at follow-up start): age group, cohort entry year, time since PC diagnosis, prior malignant neoplasm, prior secondary malignant neoplasm, prior urinary tract infection, history of hypertension, history of congestive heart failure, history of hyperlipidaemia, history of diabetes mellitus, history of renal disease, history of liver disease, prior radiation therapy, prior use of antineoplastic agents.

* P<0.05

^†^ P<0.001

**Table 3:** Total and stratified mortality and second primary malignancy rates among castration-resistant prostate cancer patients with bone metastases (mCRPC). The mCRPC cohort was too small to determine the effects of several other variables in addition to age and cohort entry year.

	Mortality			SPM		
Variable	Events	PY	IR^1^ (95% CI)	Events	PY	IR^1^ (95% CI)
**mCRPC cohort (Total)**	599	947	632.83 (584.13–685.60)	100	865	115.63 (95.05–140.67)
**Age**						
<65	128	211	606.09 (509.68–720.73)	26	191	136.20 (92.74–200.04)
65–69	126	182	693.94 (582.76–826.33)	16	175	91.56 (56.09–149.45)
70–74	130	246	528.76 (445.25–627.93)	24	230	104.22 (69.86–155.50)
75–79	118	182	647.47 (540.58–775.50)	23	158	145.90 (96.96–219.56)
80–84	63	90	697.11 (544.57–892.36)	8	85	93.59 (46.80–187.15)
>84	34	35	963.27 (688.29–1348.12)	3	26	116.41 (37.54–360.93)
**Cohort entry year**						
2006	126	237	531.6 (446.4, 633.0)	35	206	170.2 (122.2, 237.0)
2007–2009	234	360	649.9 (571.7, 738.7)	29	335	86.5 (60.1, 124.5)
2010–2013	239	349	684.0 (602.5, 776.4)	36	324	111.2 (80.2, 154.1)

^1^ Incidence rate (IR) per 1,000 patient years (PY)

During the total 32,623 and 865 person-years in the mPC and mCRPC cohorts, we observed 2,669 and 100 SPM events, respectively. The corresponding incidence rates of SPM were 81.8 (95% CI: 78.8–85.0) and 115.6 (95% CI: 95.1–140.7) per 1,000 person-years. Old age was not clearly associated with increased SPM incidence (Tables 2 and 3).

In multivariate-adjusted analyses (Table 3), high age, prior neoplasms, urinary tract infection, hypertension, congestive heart failure, diabetes, renal disease and prior use of antineoplastic agents were associated with increased mortality risk. Furthermore, prior neoplasms, liver disease and prior use of antineoplastic agents were associated with increased SPM risk (Table 3).

For the mPC cohort, the incidence rate per 1,000 person-years was 56.2 (95% CI: 53.2–58.7) for all solid tumors combined, 9.0 (95% CI: 8.0–10.0) for bladder cancer, 2.4 (95% CI: 1.9–2.9) for rectum cancer, 4.1 (95% CI: 3.4–4.8) for colon cancer, 4.2 (95% CI: 3.6–5.0) for lung cancer, 0.35 (95% CI: 0.21–0.61) for myelodysplastic syndrome, and 1.5 (95% CI: 1.2–2.0) for leukemia. As for the SPM, age was not clearly associated with these outcomes (Table C in S1 File).

For the mCRPC cohort, the incidence rate per 1,000 person-years was 82.3 (95% CI: 65.4–103.5) for all solid tumors combined, 7.5 (95% CI: 3.6–15.7) for bladder cancer, 1.1 (95% CI: 0.2–7.5) for rectum cancer, 4.3 (95% CI: 1.6–11.4) for colon cancer, 2.1 (95% CI: 0.5–8.5) for lung cancer, and 3.2 (95% CI: 1.0–9.8) for leukemia (Table D in S1 File). The number of events was 0 for myelodysplastic syndrome, and less than 5 also for all other site-specific cancers except for bladder cancer and solid tumors, not allowing further conclusions to be made.

## Discussion

We identified the mPC cohort based on recorded diagnoses of PC and bone metastases, and mCRPC cohorts using drugs indicating mCPRC (mitoxantrone, estramustine, ketoconazole, docetaxel, cabazitaxel). The identified mPC cohort was relatively old (75 years of age), while the mCRPC was younger (70 years of age). Compared to younger patients, elderly patients can be less likely to receive treatment for mCRPC, which can explain the lower age observed in this cohort. A high proportion (62%) of mPC patients had bone metastases diagnoses at the time of PC diagnosis. Compared with mPC, patients identified with mCRPC had more commonly history of prior comorbidities and therapies such as prior cancer, renal disease and prior radiation therapy. Median survival in the mPC cohort was 1.5 years, which is in line with other prior studies[14]. Patients identified with mCRPC by specific treatments lived 1.1 years on average, which is also in line with prior studies from the same time period [6,14]. In more recent clinical trials, survival has been longer, probably due to new improved therapies such as abiraterone[9,24] and enzalutamide[7]. However, our results are based on observational real-world data, and the population in such studies can considerably differ from that in clinical trials. For future studies, it may be valuable to study the effectiveness of new therapies in real-word setting. For this purpose, our study can provide valuable reference information from the preceding era.

The shorter-living mCRPC cohort was younger than mPC, our method for identifying mCRPC clearly captured more advanced patients, as should be expected. Survival time in this study was clearly heterogeneous: some died soon after cohort entry whereas some survived relatively long, and a bulk of follow-up time accumulated from those patients who survived the second year after cohort entry. This phenomenon emphasizes the importance of identifying correct therapy with best risk-benefit ratio for each individual. High age, prior neoplasms, urinary tract infection, congestive heart failure, diabetes and renal disease were most strongly associated with increased mortality risk.

To our knowledge, there are no prior published studies of SPM among mPC or mCRPC patients. We found that the SPM incidence in the mPC and mCRPC cohorts were 81.8 and 115.6 per 1,000 person years, respectively. Compared with the general population in Sweden, these seem relatively high. For a more detailed comparison to the general population, we used site-specific cancer rates, because the definition of SPM may vary across sources. Among 70–75 year old Swedish males in 2007, the incidence rate of lung cancer (as an example), was 2.1 per 1,000 years and the incidence rate of any cancer (excluding non-melanoma skin cancer, breast and prostate cancer) was 13.2 per 1,000 years[30]. Among mPC population in this study the lung cancer rate was clearly higher: 4.2 per 1,000 years. This might not be surprising, however, as even among PC patients SPM rates have been observed to be higher than in the general population in relation with radiation exposure [31,32]. Potential reasons for the increased SPM rates include true association with PC, and higher detection rate by increased diagnostic activity.

We did not observe that older patients in this study would be in a clearly higher SPM risk than younger ones. In such generally old population this result is not completely unexpected, as the same phenomenon can be observed based on general cancer statistics in the Nordic countries[30]. It is likely that diagnostic activity decreases at very old ages and reduces the number of observed cancers. In addition, factors affecting cancer rate and cumulating during age might surpass the age effect in SPM rate at very old ages.

We identified several comorbidities and therapies that were associated with high SPM rates Namely, prior neoplasms and prior use of antineoplastic agents were most strongly associated with increased SPM risk. These factors were also more prevalent in the mCRPC cohorts and might explain higher SPM rates as compared to the mPC cohort. It is therefore important to consider the baseline prevalence of these factors when making comparison across results in this study and in future studies, i.e., when using results from this study as a historical reference.

The main limitation in this study was related to the definition of mCRPC as only treatment information but no laboratory measurements indicating disease progression (e.g. PSA-values) were available. Our approach was based on treatments indicating mCRPC. Although this method should capture mCRPC patients, it has the caveat that it may also leave out several patients with CRPC, as hospital-administered therapies (docetaxel and cabazitaxel) were recorded poorly in the used databases. It seems most important to compare baseline characteristics comprehensively to ascertain comparability of this difficultly identifiable mCRPC cohort when making external comparisons across different studies. The identification of mPC was based more standardized diagnoses of PC and bone metastases.

Detailed information on prescribed medications that are dispensed by community pharmacies is available in PCBaSe but the capture on medications given in a hospital is limited. This caused under-reporting of hospital treatments such as cabazitaxel and docetaxel. In addition, we were not able to investigate chemotherapy effects on the outcomes, nor provide reliably how commonly these were used. This under-reporting could have also caused that relatively low proportion (7%) of eligible mPC patients were identified as having mCRPC. Certain other factors that may affect the incidence of developing cancer were not available for this study either, including smoking, alcohol use, obesity and other lifestyle factors. When comparing SPM rates across different studies and not accounting for these factors can cause residual confounding.

Finally, cancer risk can manifest only after a latency period ranging from few years in leukemia to over 10 years for solid tumors[33]. In studies analyzing cancer outcomes, it is therefore common to apply a latency period, at least as a sensitivity analysis. In this study, however, the survival time in mPC and mCRPC cohorts is so short that the use of latency periods that would exclude SPM events occurring immediately after cohort entry were not applicable.

## Supporting information

S1 File Table A. Detailed variable definitions. Table B. Medication use after bone metastases diagnosis in the mPC and mCRPC cohorts. Table C. Total and age-stratified incidence rates of solid tumors, bladder cancer, rectum cancer, colon cancer, lung cancer, myelodysplastic syndrome and leukemia among prostate cancer patients with bone metastases (mPC). Table D. Total and age-stratified incidence rates of solid tumors, bladder cancer, rectum cancer, colon cancer, lung cancer, myelodysplastic syndrome and leukemia among prostate cancer patients with metastatic castration resistant prostate cancer (mCRPC).
(DOCX)

S2 File Data request to PCBaSe by EPID research. (DOCX)
