# Supplementary material for: Overall survival and second primary malignancies in men with metastatic prostate cancer
Source: PLoS One. 2020 Feb 21;15(2):e0227552. doi: 10.1371/journal.pone.0227552 (PMC7034858; doi:10.1371/journal.pone.0227552)
Supplement: S1 File — Table A. Detailed variable definitions. Table B. Medication use after bone metastases diagnosis in the mPC and mCRPC cohorts. Table C. Total and age-stratified incidence rates of solid tumors, bladder cancer, rectum cancer, colon cancer, lung cancer, myelodysplastic syndrome and leukemia among prostate cancer patients with bone metastases (mPC). Table D. Total and age-stratified incidence rates of solid tumors, bladder cancer, rectum cancer, colon cancer, lung cancer, myelodysplastic syndrome and leukemia among prostate cancer patients with metastatic castration resistant prostate cancer (mCRPC). (DOCX) [file pone.0227552.s001.docx]

**Table A.**

| **Variable** |  | **Definition** | **Source registers** |
| --- | --- | --- | --- |
| PC diagnosis |  | ICD-10 codes: C61 | NPCR. |
| Bone metastasis diagnosis |  | ICD-10 codes: C79.5 | SCR, NPR, NPCR. |
| Androgen deprivation therapy (ADT) |  | ATC codes: L02BB01 (bicalutamide), L02BB02 (Flutamide), L02BB03 (Nilutamide), L02BX01 (Abarelix), L02AE01 (Buserelin), L02BX02 (Degarelix), H01CA01 (Gonadorelin), L02AE03 (Goesrelin), L02AE05 (Histerlin), L02AE02 (Leuprorelin), H01CA02 (Nafarelin), L02AE04 (Triptorelin) | SPDR. |
| Other ADT |  | ATC codes: G03HA01 (Cyproterone acetate), L02AB02 (Medroxyproge strerone acetate), L02AA02 (Polyestradiol phosphate), L02AB01 (Megestrol), L02AA01 (Diethystilbestrol) | SPDR. |
| Bilateral orchiectomy |  | NCSP codes: KFC10 | NPR. |
| Treatment for CRPC |  | ATC codes: L01DB07 (mitoxantrone), L01XX11 (estramustine), J02AB02 (ketoconazole), L01CD02 (docetaxel), L01CD04 (cabazitaxel) | SPDR. |
| Treatment for mCRPC |  | ATC codes: L02BX03 (abiraterone), L02BB04 (enzalutamide) | SPDR. |
| Radiopharmaceuticals for bone metastases |  | V10BX02 (samarium), V10BX01 (strontium), V10BX03 (rhenium), V10XX03 (radium) | SPDR. |
| Second primary malignancy (SPM) outcome |  | First record of ICD-10: C00 – C76, C81 – C96, D00 – D09, D37 – D48, excluding C61, or C40 – C41 during follow-up. First record of each ICD-10 code was ensured by verifying that no prior record existed with accuracy of 3 digits (e.g. C16.1 was counted as a first record only if the same person had no history of C16 ICD-10 codes). | SCR, NPR. |
| Solid tumour outcome |  | First record of ICD-10: C00 – C75, excluding C61 or C40 – C41 during follow-up. First record of each ICD-10 code was ensured by verifying that no prior record existed with accuracy of 3 digits (e.g. C16.1 was counted as a first record only if the same person had no history of C16 ICD-10 codes). | SCR, NPR. |
| Bladder cancer outcome |  | First record of ICD-10: C67, D09.0 during follow-up. Verifying that no prior records of the same outcome existed was used to ensure first record. | SCR, NPR. |
| Rectum cancer outcome |  | First record of ICD-10: C20 – C21 during follow-up. Verifying that no prior records of the same outcome existed was used to ensure first record. | SCR, NPR. |
| Colon cancer outcome |  | First record of ICD-10: C18 – C19 during follow-up. Verifying that no prior records of the same outcome existed was used to ensure first record. | SCR, NPR. |
| Lung cancer outcome |  | First record of ICD-10: C33 – C34 during follow-up. Verifying that no prior records of the same outcome existed was used to ensure first record. | SCR, NPR. |
| Myelodysplastic syndrome outcome |  | First record of ICD-10: D46 during follow-up. Verifying that no prior records of the same outcome exist was used to ensure first record. | SCR, NPR. |
| Leukaemia outcome |  | First record of ICD-10: C91 – C95 during follow-up. Verifying that no prior records of the same outcome existed was used to ensure first record. | SCR, NPR. |
| Malignant neoplasm comorbidity |  | ICD-10 codes: C00 – C75, excluding C44 and C61 | SCR, NPR |
| Secondary malignant neoplasm comorbidity |  | ICD-10 codes: C78, C79.0, C79.1, C79.3, C79.4 or C79.7 | SCR, NPR |
| Urinary tract infection comorbidity |  | ICD-10 codes: N39.0 | NPR. |
| Impotence comorbidity |  | ICD-10 codes: N40 | NPR. |
| Retention of urine comorbidity |  | ICD-10 codes: R33 | NPR. |
| Hyperplasia of prostate comorbidity |  | ICD-10 codes: N48.4, F52.2 | NPR. |
| Abnormal serum enzyme levels comorbidity |  | ICD-10 codes: R74 | NPR. |
| Other disorders of urinary system comorbidity |  | ICD-10 codes: N39 | NPR. |
| Renal disease comorbidity |  | ICD-10 codes: N00-N29 | NPR. |
| Hypertension comorbidity |  | ICD-10 codes: I10-I15 | NPR. |
| Liver disease comorbidity |  | ICD-10 codes: K70-K77 | NPR. |
| Angina pectoris comorbidity |  | ICD-10 codes: I20 | NPR. |
| Congestive heart failure comorbidity |  | ICD-10 codes: I50 | NPR. |
| Diabetes mellitus comorbidity |  | ICD-10 codes: E10-E14 | NPR. |
| Hyperlipidaemia comorbidity |  | ICD-10 codes: E78 | NPR. |
| Antineoplastic or immunomodulatory agent |  | ATC codes: L (all) | SPDR. |
| Endocrine therapy |  | ATC codes: L02 (all) | SPDR. |
| Radical prostatectomy |  | NCSP codes: KEC00, KEC01, KEC10, KEC20 | NPR. |
| Radiation therapy |  | NCSP codes: DV069, DV070, DV071, DV072 | NPR. |
| Prednisolone |  | ATC codes: H02AB06 | SPDR. |
| Opioids |  | ATC codes: N02A | SPDR. |

**Table B.**

| **Medication** | **mPC cohort** | **mCRPC cohort** |
| --- | --- | --- |
| **First generation antiandrogens** |  |  |
| Bicalutamide | 6,628 (41.55%) | 369 (53.25%) |
| Flutamide | 602 (3.77%) | 47 (6.78%) |
| Nilutamide | 2 (0.01%) | 1 (0.14%) |
| **Luteinizing hormone-releasing hormone (LHRH) - Agonists/Antagonists** |  |  |
| Abarelix | 0 (0.00%) | 0 (0.00%) |
| Buserelin | 1,517 (9.51%) | 88 (12.70%) |
| Degarelix | 6 (0.04%) | 0 (0.00%) |
| Gonadorelin | 0 (0.00%) | 0 (0.00%) |
| Goserelin | 2,129 (13.35%) | 189 (27.27%) |
| Histrelin | 42 (0.26%) | 1 (0.14%) |
| Leuprorelin | 5,508 (34.53%) | 334 (48.20%) |
| Nafarelin | 0 (0.00%) | 0 (0.00%) |
| Triptorelin | 83 (0.52%) | 4 (0.58%) |
| **Other identifiers for castration therapies** |  |  |
| Cyproterone acetate | 325 (2.04%) | 24(3.46%) |
| Medroxyprogesterone acetate | 82 (0.51%) | 13(1.88%) |
| Polyestradiol phosphate | 873 (5.47%) | 65(9.38%) |
| Megestrol | 0 (0.00%) | 0 (0.00%) |
| Diethylstilbestrol | 0 (0.00%) | 0 (0.00%) |
| **Treatments for advanced prostate cancer/CRPC treatments** |  |  |
| Mitoxantrone | 1 (0.01%) | 1 (0.14%) |
| Sipuleucel-T | 0 (0.00%) | 0 (0.00%) |
| Estramustine | 471 (2.95%) | 471 (67.97%) |
| Ketoconazole | 328 (2.06%) | 328 (47.33%) |
| **Chemotherapy** |  |  |
| Docetaxel | 49 (0.31%) | 49 (7.07%) |
| Cabazitaxel | 0 (0.00%) | 0 (0.00%) |
| **mCRPC treatments** |  |  |
| Abiraterone | 47 (0.29%) | 47 (6.78%) |
| Enzalutamide | 0 (0.00%) | 0(0.00%) |

**Table C.**

| **Strata** | **Events** | **Person years** | **Rate per 1000 person years with 95% CI** |  | **Events** | **Person years** | **Rate per 1000 person years with 95% CI** |
| --- | --- | --- | --- | --- | --- | --- | --- |
| **Solid tumor** | | | |  | **Bladder cancer** | | |
| **Total** | 1912 | 34,026 | 56.19 (53.19, 58.77) |  | 325 | 36,265 | 8.96 (8.04, 9.99) |
| **Age** |  |  |  |  |  |  |  |
| <65 | 279 | 4,873 | 57.26 (50.92, 64.39) |  | 59 | 5,094 | 11.58 (8.97, 14.95) |
| 65-69 | 282 | 5,095 | 55.35 (49.25, 62.20) |  | 36 | 5,378 | 6.69 (4.83, 9.28) |
| 70-74 | 366 | 6,420 | 57.01 (51.46, 63.16) |  | 63 | 6,817 | 9.24 (7.22, 11.83) |
| 75-79 | 436 | 7,291 | 59.80 (54.44, 65.68) |  | 79 | 7,801 | 10.13 (8.12, 12.63) |
| 80-84 | 334 | 6,251 | 53.44 (48.00, 59.48) |  | 53 | 6,675 | 7.94 (6.07, 10.39) |
| >84 | 215 | 4,097 | 52.48 (45.92, 59.99) |  | 35 | 4,499 | 7.78 (5.59, 10.84) |
| **Rectum cancer** | | | |  | **Colon cancer** | | |
| **Total** | 86 | 36,670 | 2.35 (1.90, 2.90) |  | 148 | 36,568 | 4.05 (3.44, 4.75) |
| **Age** |  |  |  |  |  |  |  |
| <65 | 7 | 5,155 | 1.36 (0.65, 2.85) |  | 18 | 5,140 | 3.50 (2.21, 5.56) |
| 65-69 | 9 | 5,463 | 1.65 (0.86, 3.17) |  | 12 | 5,443 | 2.20 (1.25, 3.88) |
| 70-74 | 19 | 6,890 | 2.76 (1.76, 4.32) |  | 32 | 6,868 | 4.66 (3.30, 6.59) |
| 75-79 | 20 | 7,889 | 2.54 (1.64, 3.93) |  | 41 | 7,862 | 5.21 (3.84, 7.08) |
| 80-84 | 19 | 6,729 | 2.82 (1.80, 4.43) |  | 26 | 6,714 | 3.87 (2.64, 5.69) |
| >84 | 12 | 4,545 | 2.64 (1.50, 4.65) |  | 19 | 4,542 | 4.18 (2.67, 6.56) |
| **Lung cancer** | | | |  | **Myelodysplastic syndrome** | | |
| **Total** | 155 | 36,682 | 4.23 (3.61, 4.95) |  | 13 | 36,790 | 0.35 (0.21, 0.61) |
| **Age** |  |  |  |  |  |  |  |
| <65 | 21 | 5,151 | 4.08 (2.66, 6.25) |  | 1 | 5,165 | 0.19 (0.03, 1.37) |
| 65-69 | 33 | 5,459 | 6.05 (4.30, 8.50) |  | 1 | 5,470 | 0.18 (0.03, 1.30) |
| 70-74 | 29 | 6,877 | 4.22 (2.93, 6.07) |  | 4 | 6,906 | 0.58 (0.22, 1.54) |
| 75-79 | 37 | 7,895 | 4.69 (3.40, 6.47) |  | 1 | 7,916 | 0.13 (0.03, 0.90) |
| 80-84 | 24 | 6,741 | 3.56 (2.39, 5.31) |  | 3 | 6,765 | 0.44 (0.14, 1.37) |
| >84 | 11 | 4,560 | 2.41 (1.34, 4.36) |  | 3 | 4,568 | 0.66 (0.21, 2.04) |
| **Leukemia** |  |  |  |  |  |  |  |
| **Total** | 56 | 36,733 | 1.52 (1.17, 1.98) |  |  |  |  |
| **Age** |  |  |  |  |  |  |  |
| <65 | 9 | 5,156 | 1.75 (0.91, 3.35) |  |  |  |  |
| 65-69 | 6 | 5,468 | 1.10 (0.49, 2.44) |  |  |  |  |
| 70-74 | 10 | 6,902 | 1.45 (0.78, 2.69) |  |  |  |  |
| 75-79 | 12 | 7,905 | 1.52 (0.86, 2.67) |  |  |  |  |
| 80-84 | 12 | 6,744 | 1.78 (1.01, 3.13) |  |  |  |  |
| >84 | 7 | 4,558 | 1.54 (0.73, 3.22) |  |  |  |  |

**Table D.**

| **Strata** | **Events** | **Person years** | **Rate per 1000 person years with 95% CI** |  | **Events** | **Person years** | **Rate per 1000 person years with 95% CI** |
| --- | --- | --- | --- | --- | --- | --- | --- |
| **Solid tumor** | | | |  | **Bladder cancer** | | |
| **Total** | 73 | 887 | 82.29 (65.42, 103.51) |  | 7 | 938 | 7.46 (3.56, 15.66) |
| **Age** |  |  |  |  |  |  |  |
| <65 | 18 | 196 | 91.74 (57.8, 145.61) |  | 2 | 209 | 9.58 (2.4, 38.32) |
| 65-69 | 12 | 179 | 67.21 (38.17, 118.35) |  | 0 | 182 | NA (NA, NA) |
| 70-74 | 20 | 233 | 85.92 (55.43, 133.18) |  | 2 | 245 | 8.16 (2.04, 32.62) |
| 75-79 | 14 | 167 | 83.99 (49.74, 141.81) |  | 2 | 178 | 11.22 (2.81, 44.87) |
| 80-84 | 7 | 86 | 81.56 (38.88, 171.09) |  | 1 | 89 | 11.2 (1.58, 79.5) |
| >84 | 2 | 27 | 73.86 (18.47, 295.3) |  | 0 | 35 | NA (NA, NA) |
| **Rectum cancer** | | | |  | **Colon cancer** | | |
| **Total** | 1 | 946 | 1.06 (0.15, 7.5) |  | 4 | 936 | 4.27 (1.6, 11.39) |
| **Age** |  |  |  |  |  |  |  |
| <65 | 0 | 211 | NA (NA, NA) |  | 0 | 211 | NA (NA, NA) |
| 65-69 | 0 | 182 | NA (NA, NA) |  | 0 | 182 | NA (NA, NA) |
| 70-74 | 0 | 246 | NA (NA, NA) |  | 1 | 245 | 4.08 (0.57, 28.96) |
| 75-79 | 1 | 182 | 5.5 (0.77, 39.04) |  | 2 | 180 | 11.12 (2.78, 44.45) |
| 80-84 | 0 | 90 | NA (NA, NA) |  | 0 | 89 | NA (NA, NA) |
| >84 | 0 | 35 | NA (NA, NA) |  | 1 | 29 | 33.98 (4.79, 241.22) |
| **Lung cancer** | | | |  | **Myelodysplastic syndrome** | | |
| **Total** | 2 | 944 | 2.12 (0.53, 8.47) |  | 0 | 947 | NA (NA, NA) |
| **Age** |  |  |  |  |  |  |  |
| <65 | 0 | 211 | NA (NA, NA) |  | 0 | 211 | NA (NA, NA) |
| 65-69 | 0 | 182 | NA (NA, NA) |  | 0 | 182 | NA (NA, NA) |
| 70-74 | 1 | 245 | 4.08 (0.57, 28.96) |  | 0 | 246 | NA (NA, NA) |
| 75-79 | 1 | 180 | 5.55 (0.78, 39.42) |  | 0 | 182 | NA (NA, NA) |
| 80-84 | 0 | 90 | NA (NA, NA) |  | 0 | 90 | NA (NA, NA) |
| >84 | 0 | 35 | NA (NA, NA) |  | 0 | 35 | NA (NA, NA) |
| **Leukemia** |  |  |  |  |  |  |  |
| **Total** | 3 | 946 | 3.17 (1.02, 9.84) |  |  |  |  |
| **Age** |  |  |  |  |  |  |  |
| <65 | 0 | 211 | NA (NA, NA) |  |  |  |  |
| 65-69 | 0 | 182 | NA (NA, NA) |  |  |  |  |
| 70-74 | 1 | 246 | 4.07 (0.57, 28.88) |  |  |  |  |
| 75-79 | 1 | 182 | 5.5 (0.77, 39.04) |  |  |  |  |
| 80-84 | 1 | 90 | 11.13 (1.57, 79.03) |  |  |  |  |
| >84 | 0 | 35 | NA (NA, NA) |  |  |  |  |
