# Supplementary material for: Overall survival and second primary malignancies in men with metastatic prostate cancer
Source: PLoS One. 2020 Feb 21;15(2):e0227552. doi: 10.1371/journal.pone.0227552 (PMC7034858; doi:10.1371/journal.pone.0227552)
Supplement: S2 File — (DOCX) [file pone.0227552.s002.docx]

**Bilaga 4. List of variables from PCBaSe, by source register.**

The study population includes all men who between 1^st^ January 1998 and 31^st^ December 2013 were registered in The National Prostate Cancer Register (NPCR) of Sweden. The NPCR has a capture rate of 98% compared to the Swedish Cancer Registry to which registration is mandated by law.

The NPCR includes a comprehensive data set of cancer characteristics at date of diagnosis including information of serum levels of prostate specific antigen (PSA), and TNM status including bone imaging for assessment of bone metastases. The NPCR also holds and data on primary cancer treatment, prostate cancer diagnosis (ICD-10 code C61) and a diagnosis for bone metastases (ICD-10 code C79.5) and/or bone-directed treatments for bone metastases.

The Prescribed Drug Register which started on July 2005 includes all filled prescriptions in the outpatient setting, but does not hold information on drugs that are delivered by a department either in-patient or out-patient. Thus, the register does not include chemotherapy delivered at a department. As the study period of this study ends in 2013, filled prescriptions for novel antiandrogens abiraterone and enzalutamide, which came in operation in July 2015, are also not captured from the Prescribed Drug Register.

Variables are extracted for the study population from the time period of 1^st^ January 1998 – 31^st^ December 2013 (as available). A patient identifier shall be available for all data.

**From the National Prostate Cancer Register (NPCR)**

| **Analysis variable / purpose** | **Formulär 1**  **kortnamn** | **Formulär 2**  **kortnamn** | **Formulär 3**  **kortnamn** |
| --- | --- | --- | --- |
| Defining age of the included individuals^[[1]](#footnote-1)^ | From variables:  D_Rappdat  🡪  1) create age (in years) at reporting date  1) create age (in years) 1^st^ January of the reporting year | From variables:  D_Rappdat  🡪  1) create age (in years) at reporting date  1) create age (in years) 1^st^ January of the reporting year | From variables:  D_Rappdat  🡪  1) create age (in years) at reporting date  1) create age (in years) 1^st^ January of the reporting year |
| Date information for diagnoses, operations, measurements etc. to determine when event happened. | D_DiaDat, D_Rappdat (backup) | B_BehBesDat, B_RappDat (backup) | s_stralanmdat, s_stratdatrt, s_startdatboost, s_startdatseeds, s_rappdat (backup) |
| PSA-value for population description at baseline and follow-up to investigate possible increases | D_SPSA |  | S_SPSA |
| Prostate volume for population description at baseline | D_Vol |  |  |
| TNM-staging for population description at baseline | D_Tstad, D_Nstad, D_Mstad |  |  |
| Gleason score for population description at baseline | D_GleasEtt, D_GleasTva, D_GleasSA |  |  |
| WHO Differentiation for population description at baseline | D_Diffgrad |  |  |
| Biopsy variables for population description at baseline | D_Biop, D_BiopCa, D_mmcancer, D_bioplangd |  |  |
| Type of treatments given to support exposure definitions |  | B_BehTyp,  B_KonsTer,  B_RT,  B_PallKirKast,  B_PallGnRH, B_PallAntiand, B_PallOvrHorm, B_PallOrvHormTxt, B_PallBisfos, B_PallAnnBeh, B_PallAnnBehTxt, B_PallOstr,  B_AnnBeh,  B_KurHorm, B_KurHormBeh, B_Adjlngen,  B_AdjExtRT,  B_adjrtremtill, B_adjrtremtillkod, B_adjrtremtillkod, B_AdjGnRH,  B_AdjAntiand,  B_AdjCyt | s_primextrt, s_postop_rt, s_TidAnnPrimbeh, s_boost, s_seeds, s_neoadjhormbeh, hormbeh1, s_hormbehtyp1, s_hormbehlangd1, s_hormbeh2, s_hormbehtyp2, s_hormbehlangd2 |

**From the National Patient Register, outpatients** (dataset “par_ov”), all healthcare encounters and related information.

| **Dataset** | **Variabelnamn** | **Definition** | **Use in analyses (purpose)** |
| --- | --- | --- | --- |
| par_ov | DIAGNOS | Diagnoser enligt ICD10. Max 30 diagnoser | To determine patient population, cohort entry date, comorbidities and other variables. Will be used to describe the population and in stratified analyses. |
| par_ov | HDIA | Angiven huvuddiagnos | To determine patient population, cohort entry date, comorbidities and other variables. Will be used to describe the population and in stratified analyses. |
| par_ov | INDATUM | Datum för öppenvårdkontakt. Numerisk | To determine diagnosis, treatment or procedure date. Will be used to define outcomes, cohort entry and several co-variables. |
| par_ov | INDATUMA | Datum för öppenvårdkontakt. Text | To determine diagnosis, treatment or procedure date. Will be used to define outcomes, cohort entry and several co-variables. |
| par_ov | KON | Patientens kön | Check population that all are male |
| par_ov | LKF | Patientens hemort efter kontroll mot SCB | To determine county-level region of residence (Swedish län/landsting), which is used in stratified analyses. |
| par_ov | OP | Åtgärdkoder max 30 åtgärder | To determine treatment procedures. Used e.g. in the definition of sub-populations and in stratified analyses. |

**From the National Patient Register, inpatients** (dataset “par_sv”), all healthcare encounters and related information.

| **Dataset** | **Variabelnamn** | **Definition** | **Use in analyses (purpose)** |
| --- | --- | --- | --- |
| par_sv | DIAGNOS | Diagnoser enligt ICD10 max 30 koder | To determine patient population, cohort entry date, comorbidities and other variables. Will be used to describe the population and in stratified analyses. |
| par_sv | HDIA | Angiven huvuddiagnos | To determine patient population, cohort entry date, comorbidities and other variables. Will be used to describe the population and in stratified analyses. |
| par_sv | INDATUM | Det datum då patienten skrivs in. Antal dagar från år 1960-01-01 fram t.o.m. INDATUM | To determine diagnosis, treatment or procedure date. Will be used to define outcomes, cohort entry and several co-variables. |
| par_sv | INDATUMA | Det datum då patienten skrivs in | To determine diagnosis, treatment or procedure date. Will be used to define outcomes, cohort entry and several co-variables. |
| par_sv | KON | Patientens kön | Check population that all are male |
| par_sv | LKF | Patientens hemort efter kontroll mot SCB | To determine county-level region of residence (Swedish län/landsting), which is used in stratified analyses. |
| par_sv | OP | Åtgärdkoder max 30 åtgärder | To determine treatment procedures. Used e.g. in the definition of sub-populations and in stratified analyses. |
| par_sv | UTDATUM | Det datum då patienten skrivs ut | To determine days spent in hospital |
| par_sv | UTDATUMA | Det datum då patienten skrivs ut | To determine days spent in hospital |

**From the Swedish Cancer Registry**, all diagnosed cancers and related information.

| **Variabelnamn** | **Definition** | **Use in analyses (purpose)** |
| --- | --- | --- |
| BEN | Indikator för malign eller benign tumör enligt morfologi. | To allow exclusion of benign tumors. |
| DIADAT | Datum då diagnosen fastställdes | To determine time with and without specific cancers. Used to define outcomes. |
| DIADATN | Datum då diagnosen fastställdes | To determine time with and without specific cancers. Used to define outcomes. |
| DIGR | Typ av undersökning som låg till grund för diagnosen. Se BilagaCAN4.doc | Population description |
| ICD9 | Tumörens lokalisation enligt ICD-9 | To determine time with and without specific cancers. Used to define outcomes. |
| ICDO10 | Tumörens lokalisation enligt ICD-O/2 (med vissa inslag av ICD-10) | To determine time with and without specific cancers. Used to define outcomes. |
| ICDO3 | Tumörens lokalisation enligt ICD-O/3 | To determine time with and without specific cancers. Used to define outcomes. |
| KON | Patientens kön | Check population that all are male |
| LKF | Patientens folkbokföringsort vid diagnostillfället | To determine county-level region of residence (Swedish län/landsting), which is used in stratified analyses. |
| M | Tumörutbredning vid diagnostillfället, fjärrmetastaser. | For description of PC population and to support diagnosis and TNM staging information. |
| N | Tumörutbredning vid diagnostillfället, lymfkörtlar. | For description of PC population and to support diagnosis and TNM staging information. |
| PAD | Tumörens histopatologiska diagnos enligt C24.1, se BilagaCAN3.doc | Population description |
| SNOMED3 | Tumörens morfologiska diagnos enligt ICD-O/3 | To determine time with and without specific cancers. Used to define outcomes. |
| SNOMEDO10 | Tumörens morfologiska diagnos enligt ICD-O/2 | To determine time with and without specific cancers. Used to define outcomes. |
| T | Tumörutbredning vid diagnostillfället, tumörens storlek. | For description of PC population and to support diagnosis and TNM staging information. |
| TNMGRUND | Undersökning som låg till grund för TNM. Patologisk anges om någon av T, N eller M grundas på morfologisk undersökning. Klinisk anges då endast klinisk utredning legat till grund och morfologisk undersökning saknas. | Population description |
| TNR | Tumörnummer i kronologisk ordning, samtliga rapporteringspliktiga tumörer. (Om patienten diagnostiserats med flera tumörer) | To support information about have other cancers been diagnosed before PC. |
| TNRMAL | Tumörnummer i kronologisk ordning, enbart maligna tumörer. (Om patienten diagnostiserats med flera tumörer) | To support information about have other cancers been diagnosed before PC. |

**From the Swedish Prescribed Drug Register**, specified drug purchases (see ATC-code list below) and related information.

| **Variabelnamn** | **Definition** | **Use in analyses (purpose)** |
| --- | --- | --- |
| EDATUM | Expeditionsdatum. Datum när patienten köpte varan | To determine treatment start date that is used e.g. in sub-cohort formation and in stratified analyses. |
| FDATUM | Förkskrivningsdatum. Datum när receptet är utfärdat. | To determine treatment start date (if purchase date missing) that is used e.g. in sub-cohort formation and in stratified analyses. |
| ANTAL | Antal förpackningar som har hämtats ut | To determine treatment duration that is used e.g. in sub-cohort formation and in stratified analyses. |
| VARUNR | Expedierat varunummer | To specify treatment type. |
| FVARUNR | Förskrivet varunummer, om annat än det expedierade | To specify treatment type (if varunr missing). |
| ATC | ATC-kod enligt WHO. ATC-koden klassificerar läkemedlen efter användningsområde och kemisk substans. | To determine type of treatment that is used e.g. in sub-cohort formation and in stratified analyses. |
| FORPDDD | Förpackningens DDD. Anger hur många Definerade DygnsDoser förpackningen innehåller. DDD är den genomsnittliga dygnsdosen då läkemedlet används av en vuxen vid medlets huvudindikation | To determine treatment duration that is used e.g. in sub-cohort formation and in stratified analyses |
| FORPS | Förpackningsstorlek. En sammansatt beskrivning av förpackningsstorleken, t.ex. 100x1 tablett(er), 100 ml, eller 500 kapsel/kapslar | To support definition of treatment duration. |
| STYRKALF | Anger styrkan för produkten i alfanumerisk form, t.ex. 5 mg, 9mg/ml eller 2%. | To support definition of treatment duration if DDD is missing. |
| STYRKNUM | Anger styrkan för produkten i numerisk form (utan enhet). | To support definition of treatment duration if DDD is missing. |
| STYRKAENHET | Anger enheten på styrkan för produkten, t.ex. mg, mg/ml eller %. | To support definition of treatment duration if DDD is missing. |
| LFORM | Läkemedelsform/beredningsform, t.ex. filmdragerad tablett, gel, eller inhalationspulver. | To support definition of treatment duration if DDD is missing. |

**List of ATC-codes extracted from the PCBaSe, as available (from the Swedish Prescribed Drug Register).**

| **Substance** | **ATC code** |
| --- | --- |
| Flutamide | L02BB01 |
| Nilutamide | L02BB02 |
| Bicalutamide | L02BB03 |
| Buserelin | L02AE01 |
| Leuprorelin | L02AE02 |
| Goserelin | L02AE03 |
| Triptorelin | L02AE04 |
| Histrelin | L02AE05 |
| Abarelix | L02BX01 |
| Degarelix | L02BX02 |
| Gonadorelin | H01CA01 |
| Nafarelin | H01CA02 |
| Cyproterone acetate | G03HA01 |
| Medroxyprogesterone acetate | L02AB02 |
| Polyestradiol phosphate | L02AA02 |
| Megestrol | L02AB01 |
| Diethylstilbestrole | G03CB02, L02AA01 |
| Estramustine | L01XX11 |
| Ketoconazole | J02AB02 |
| Docetaxel | L01CD02 |
| Cabazitaxel | L01CD04 |
| Mitoxantrone | L01DB07 |
| Enzalutamide | L02BB04 |
| Abiraterone | L02BX03 |
| Clodronate | M05BA02 |
| Pamidronate | M05BA03 |
| Aldendronic acid | M05BA04 |
| Ibandronic acid | M05BA06 |
| Risendronic acid | M05BA07 |
| Zoledronic acid | M05BA08 |
| Denosumab | M05BX04 |
| Radiopharmaceuticals | V10 (all in the class) |
| Dexamethsone | H02AB02 |
| Prednisolone | H02AB06 |
| Prednisone | H02AB07 |
| NSAID | M01A (all in the class) |
| Other analgetics and antipyretics | N02B (all in the class) |
| Opioids | N02A (all in the class) |
| Chemotherapy | L01 (all in the class) |

**From the Cause of Death Register**, all times of death and related information.

| **Dataset** | **Variabelnamn** | **Definition** | **Use in analyses (purpose)** |
| --- | --- | --- | --- |
| dors | DODSDAT | Den avlidnes dödsdatum | To investigate all-cause mortality outcome. |
| dors | DODSDATN | Den avlidnes dödsdatum | To investigate all-cause mortality outcome. |

**From the population register (Swedish: Registret över Totalbefolkningen, Statistiska centralbyrån)**, all dates of immigration and emigration.

| **Variable** | **Use in the analyses (purpose)** |
| --- | --- |
| All dates of emigration | To exclude from analysis time periods when patient not living in Sweden. |
| All dates of immigration | To exclude from analysis time periods when patient not living in Sweden. |

1. Dialogue with PCBaSe requested for defining the age of the included individuals. [↑](#footnote-ref-1)
